# A Practical Approach in Refining Binary Outcome for Treatment Effect of COVID-19 According to Geographical Diversity

**DOI:** 10.3390/tropicalmed8020083

**Published:** 2023-01-26

**Authors:** I-Shiang Tzeng

**Affiliations:** Department of Research, Taipei Tzu Chi Hospital, Buddhist Tzu Chi Medical Foundation, New Taipei City 23142, Taiwan; istzeng@gmail.com

**Keywords:** COVID-19, efficacy, safety, Lopinavir/Ritonavir, small number of studies bias, geographical diversity, meta-analysis

## Abstract

The recent COVID-19 pandemic has drawn attention to health and economics worldwide. Initially, diseases only ravage local populations, while a pandemic could aggravate global economic burdens. Lopinavir/Ritonavir is an anti-HIV drug that was used on small scale patients during SARS, but its effectiveness for COVID-19 treatment is still unclear. Previous studies or meta-analysis have retrieved clinical data of subgroup analysis to evaluate the efficacy and safety of Lopinavir/Ritonavir for the treatment of COVID-19 in a few affected regions. However, geographical diversity and small number of studies bias correction were not achieved in such subgroup analysis of published meta-analysis. The present study demonstrates a practical approach in refining the binary outcome for COVID-19 treatment of Lopinavir/Ritonavir according to geographical location diversity and small number of studies (less than or equal to five) for subgroup analysis. After performing practical approach, the risk of adverse event with LPV/RTV for treatment of COVID-19 becomes nonsignificant compared to previous meta-analysis. Furthermore, we also notice heterogeneity of random effect of meta-analysis may be declined after proposed adjustment. In conclusion, proposed practical approach is recommend for performing a subgroup analysis to avoid concentration in a single geographical location and small number of studies bias.

## 1. Introduction

The recent COVID-19 pandemic has drawn attention to health and economics worldwide. Not only may diseases ravage local populations, but they may also aggravate global economic burdens. Previous studies have concentrated on investigating the epidemiological impact of the COVID-19 pandemic [1,2]. For the clinical prognosis of patients with COVID-19 and organ-specific metabolic conditions, severity and mortality should be approached with caution [3,4]. Major findings have shown that there is an increased risk of the severity and mortality of liver diseases and chronic kidney disease in patients with COVID-19. A previous meta-analysis integrally assessed the prevalence of HIV in patients with COVID-19 in several regions [5] but there existed concerns of geospatial epidemiology. For example, geographical location had an effect on the prevalence and prognosis of living with HIV for patients with COVID-19 [5]. This phenomenon may result from socioeconomic inequalities in health. Consequence of socioeconomic inequalities in health showed that HIV infection may have an influence on the risk of severe COVID-19 and the likelihood of death among the global and continental regions [5]. Thus, to perform a subgroup analysis on continental regions may investigate whether inequalities in health are associated with geographical location [5].

In fact, a subgroup analysis may ignore the small number of studies bias while overestimating the pooling effect in meta-analyses [6]. The pooling-effects (i.e., random-effects model) for meta-analysis were generally adopted to integrate different effect size estimates with underlying heterogeneous true effect sizes [7,8]. The average effect and the variance among studies are generally estimated under the random-effects model. When adopting conventional DerSimonian-Laird (DL) method [9] for random-effects model, the variance among studies was estimated initially. Next was to cope with the sampling variances among studies and merged into the study weights while the average effect being estimated. This conventional protocol for conducting meta-analyses provides a pooled effect under weighted average approach. However, estimates of the variance among studies may be inexactitude especially if the small number of studies included in a meta-analysis [10,11,12]. Such uncertainty was disregarded to infer the random-effects based on a conventional normal approximation which may affect inference of accuracy. Critical concerns about the estimate of variances among studies (affect estimated effects) were being inexactitude while the studies are small. Neglect this uncertainty during integrating the random-effects which regard detrimental consequences for statistical inferences [13,14,15,16,17]. For aforementioned concern, the advantage of the applied HK adjusted method may have a more accurate of estimated random-effects and its confidence interval (CI) compared to DL approach. Correction through multiply the conventional variance of the estimated average effect with a scaling factor can tackle small number of studies size bias [18]. A previous meta-analysis compared Lopinavir/Ritonavir (LPV-RTV) and conventional therapy (no antiviral treatment), but it was only limited to a single country. The weight brought to countries specificity may decline heterogeneity on estimation of random effects compared to without using weighted approach. Due to the lack of geographical location diversity and knowledge of small number of studies bias adjustment, this study aimed to apply a geographically weighted (GW) and HK adjusted approach to refine the binary outcome for treatment effect according to geographical diversity.

## 2. Materials and Methods

### 2.1. Objective Comparisons

I adopted the binary outcome for treatment effect from a previous meta-analysis [6]. The specific items extracted from the relevant studies were as follows: first author’s surname; the small studies size criterion was number of studies less than or equal to five. A list of adjusted items was compiled as follows: (1) time to body temperature normalization (days) (LPV/RTV vs. umifenovir) [19,20]; (2) time to body temperature normalization (days) LPV/RTV vs. no antiviral treatment (conventional) [19,20]; (3) rate of cough alleviation after 7 days of treatment (LPV/RTV vs. umifenovir) [19,20]; (4) rate of cough alleviation after 7 days of treatment (LPV/RTV vs. no antiviral treatment) [19,20]; (5) rate of improvement on chest computed tomography (CT) after 7 days of treatment (LPV/RTV vs. umifenovir) [19,20]; (6) rate of improvement on chest CT after 7 days of treatment (LPV/RTV vs. no antiviral treatment or conventional) [19,20]; (7) rate of adverse events of treatment (LPV/RTV vs. umifenovir) [19,20,21]; and (8) rate of adverse events of treatment (LPV/RTV vs. no antiviral treatment or conventional) [19,20,21]. Detail of description referred to previous study [6].

### 2.2. Adjusted Analysis

The geographical diversity is the collection of distinct physical, human and cultural components that coexist in a single, comparatively limited geographic area and are found in the same zone, region or nation. I adopted different number of populations for included studies to adjust geographical diversity (refer to Supplementary Table S1). Briefly, number of population of each study location (of studies for above 8 adjusted items) can be treated as initial weight to adjust odds ratios of included studies. Formula of odds ratios was added as below:

$$\mathrm{Odds}\;\mathrm{ratio}=\frac{p_{1}/\left(1-p_{1}\right)}{p_{2}/\left(1-p_{2}\right)}$$

where $p_{1}\;$ denoted as probability of an event will occur in exposed group and $p_{2}\;$ denoted as probability of an event will occur in unexposed group. Statistical analyses were performed using R 4.2.0 software. The implementation of package “metafor” [22] was completed using R software for an adjustment analysis. DL conventional and HK adjusted approaches were augment options embed in package “metafor”. Initially, I performed DL conventional approach to confirm random-effects concise to results of previous meta-analysis [6]. Next, HK adjusted approaches [18] were performed to contrast previous results conducted by DL method [9]. Finally, a z-test is used in hypothesis testing to evaluate whether overall mean of true effect was equal to zero under random-effects model. Another statistics I^2^ was used to confirm whether there is heterogeneity among the recruited studies in subgroup meta-analysis. A *p*-value is less than 0.05 is considered to be statistically significant.

## 3. Results

I used random-effects models with GW and HK adjustment [18] for all meta-analyses of binary outcome with a small number of studies [6]. I summarized and compared odds ratio (OR) of DL method and HK adjusted method for the treatment effect (Table 1). These effects were still insignificant in the random-effects models after adapting GW and HK adjustment, except for the rate of adverse events of treatment (for LPV/RTV vs. umifenovir and LPV/RTV vs. no antiviral treatment or conventional).

In previous results, rate of adverse events of treatment in the LPV/RTV arms compared to the no antiviral treatment or conventional arms (OR = 2.66; 95% CI, 1.36 to 5.19; *p* = 0.004). After GW and HK adjustment, that of overall effect was not significant even a greater number of adverse events were reported in the LPV/RTV arms compared to the no antiviral treatment or conventional arms (OR = 0.83; 95% CI, 0.23 to 3.03; *p* = 0.60 for objective (7) in Table 1). Note that, I^2^ was 0% before adjustment and keep being 0% after adjustment. Next, the adjusted results of rate of adverse events of treatment in LPV/RTV vs. no antiviral treatment or conventional was similar to above report (OR = 1.51; 95% CI, 0.42 to 5.46; *p* = 0.30 for objective (8) in Table 1). Additionally, I^2^ from 18% before adjustment decreased to 0% after adjustment.

**Figure 1:**
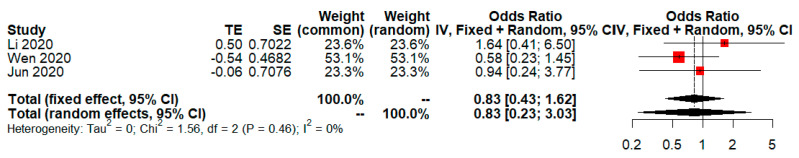
Adjusted random effects of LPV/RTV vs. umifenovir for rate of adverse events. A total of 45 adverse events were reported in the LPV/RTV arms compared to a total of 14 adverse events found in the umifenovir group and there is 0.83 times the odds, but no significance for adverse events. CI, confidence interval; df, degrees of freedom [19,20,21].

**Figure 2:**
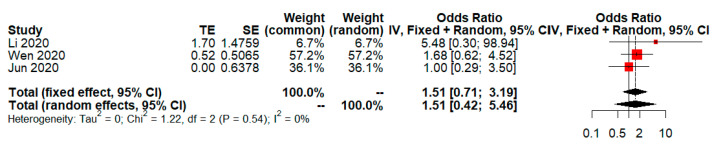
Adjusted random effects of LPV/RTV vs. no antiviral treatment or conventional for rate of adverse events. A total of 45 adverse events were reported in the LPV/RTV arms compared to the total of 10 presented in no antiviral treatment or conventional arms group and there is 1.51 times the odds, but no significance for adverse events. CI, confidence interval; df, degrees of freedom [19,20,21].

I also noticed that CI became dramatically wider after adjustment especially for (2), (3) and (4) objectives. The rest of objectives had minor or moderate inflation on CI of random effects. The results show that there was no significant difference in the rate of adverse events of treatment between the intervention and control groups after GW and HK adjustment (see Figure 1 and Figure 2).

## 4. Discussion

LPV/RTV is an anti-HIV drug that was used on small scale patients during SARS, but its effectiveness is still unclear. However, the considering that early negative and conflicting results were urgent, a systematic review and meta-analysis for the efficacy and safety of this COVID-19 treatment can tackle above concern. Although the previously conducted meta-analysis included 14 articles (a total of 9036 patients) relating to the efficacy and safety of LPV/RTV in patients with COVID-19 [6], a subgroup analysis was deemed necessary for the estimation of random effects according to few studies recruited. Geographical location diversity and small number of studies bias correction (i.e., GW and HK adjustment, respectively) were not achieved in these subgroup studies. For example, two studies recruited for integrated effect on time to temperature normalization for LPV/RTV arm vs. umifenovir arm [19,20]. In addition, a few studies recruited for integrated effect on rate of adverse events of treatment (LPV/RTV vs. umifenovir or no antiviral treatment or conventional) [19,20,21]. A few studies recruited for subgroup analysis [19,20,21] may have aforementioned drawbacks.

Regarding safety, the previous meta-analysis found that greater adverse events were reported in the LPV/RTV arm than in the no antiviral treatment (conventional) and umifenovir arms. Adverse events may be associated with LPV/RTV alone or in combination with other medicines that patients with COVID-19 reported using. In clinical practice, I attributed these clinical events to being gastrointestinal (GIT) in nature, including nausea, vomiting, and diarrhea [20]. Furthermore, serious GIT adverse drug reactions involved acute gastritis, GIT bleeding and acute kidney injury [20]. After geographical location and small number of studies bias correction, a trend was observed for adverse events, but it did not achieve statistical significance.

**Figure 3:**
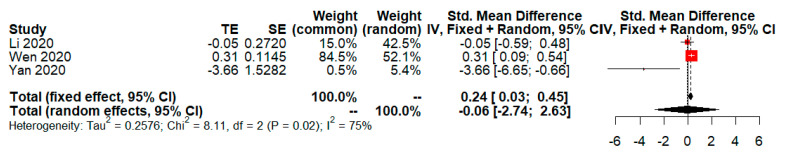
Adjusted random effects of mean difference in time from +ve to −ve PCR (days) between LPV/RTV vs. no antiviral treatment or conventional. A total of 288 patients reported on virological cure in LPV/RTV alone arm and conventional arm on day 7 among three studies. Nonsignificant mean difference (*p* = 0.93) was observed between the two arms in terms. CI, confidence interval; df, degrees of freedom [19,20,23].

**Figure 4:**
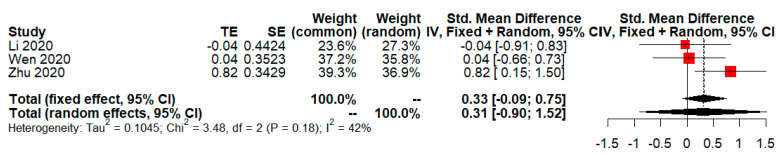
Adjusted random effects of mean difference in time from +ve to −ve PCR (days) between LPV/RTV vs. umifenovir. A total of 214 patients presented on virological cure in LPV/RTV alone arm and umifenovir arm on day 7 among three studies. No significant mean difference (*p* = 0.39) was found between the two arms in terms of virological cure. CI, confidence interval; df, degrees of freedom [19,20,24].

**Figure 5:**
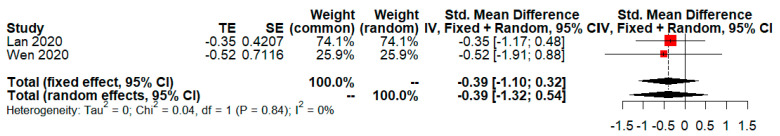
Adjusted random effects of mean difference in time from +ve to −ve PCR (days) between LPV/RTV vs. LPV/RTV plus umifenovir combination. A total of 168 patients reported on virological cure in LPV/RTV alone arm and umifenovir plus LPV/RTV arm on day 7 among two studies. No significant mean difference (*p* = 0.11) was reported between the two arms in terms of virological cure. CI, confidence interval; df, degrees of freedom [19,20].

Furthermore, GW and HK adjusted approach also was applicable for difference in continuous measurement. Mean time difference of virological cure between LPV/RTV and antiviral treatment or conventional (or umifenovir; or combination) had been investigated by previous meta-analysis [6]. Short of mean time in days for LPV/RTV arm compared with otherwise therapy aggregated with relevant studies [19,20]. After 7 days of treatment, it was discovered that the LPV/RTV arm had fewer cough days overall than the umifenovir arm or the conventional (no antiviral therapy) arm. However, it was determined that the effect was not controversial overall. In previous study, Yan et al. presented time from +ve to −ve PCR (days) had significant difference with LPV/RTV vs. no antiviral treatment or conventional [23]. Furthermore, Zhu et al. reported average PCR negative conversion times had no significant difference among interferon plus LPV/RTV or interferon plus LPV/RTV plus ribavirin treatment arms [24]. Additionally, Lsn et al. found that time from +ve to −ve PCR (days) had no significant difference with LPV/RTV vs. LPV/RTV plus umifenovir combination [25]. The results of the overall effect were not significant. I also had adjusted effects on mean time difference of virological cure between LPV/RTV and antiviral treatment or conventional (or umifenovir; or combination, respectively). Figure 3, Figure 4 and Figure 5 showed insignificant effects after adjustment. Notice that, CI of random effects existed huge length in Figure 1 and Figure 2. I may attribute to power decline for the relevant test [26]. According to previous study, weighted modification estimation also had short width of CI [27].

There are several limitations of this study, including a few clinical studies investigating the efficacy and safety of LPV/RTV in combination with small number of studies of participants. Overall, as the number of studies was small and many test completed likely were skewed due to low number of cases. In the meantime, subgroup analysis of treatment effects limited on single country (i.e., violation of geographical diversity). Another limitation is overall random effects may be opposite because different methodology used to perform meta-analysis specifically for the results of efficacy and safety of using LPV/RTV in combination with other agents versus no antiviral therapy (conventional therapy) or control. To our knowledge, the HK approach was equivalent to the weighted least squares regression models under the assumption of error variances had be known [28]. Last limitation for the adjusted ORs was inflation may be caused by spare effect [29]. Aggregate above limitations, this study demonstrates a practical approach to refine binary outcome for treatment of the efficacy, safety and clinical outcomes of LPV/RTV alone or with other antiviral medications.

## 5. Conclusions

The subgroup analysis included small number of studies in this study and found that no statistical evidence advantage in the efficacy of LPV/RTV in COVID-19 patients. Based on its easy application, the practical approach proposed in this study to refine the binary outcome for treatment effect according to geographical diversity is a good alternative to the tests performed in subgroup analysis studies. The most important finding in this study was risk of the adverse event with LPV/RTV for treatment of COVID-19 becomes nonsignificant after adjustment. Furthermore, we also notice heterogeneity of random effect of meta-analysis may be declined (i.e., based on objective (8) analysis results) after proposed adjustment. In summary, integrate GW and HK adjustment method is recommend for performing a subgroup analysis to avoid concentration in a single geographical location and small number of studies bias.

## Figures and Tables

**Table 1:** Summary of unadjusted and adjusted random effects for comparisons between LPV/RTV and other treatments.

Objectives or Intervention vs. Control	Included Studies (Year, Country)	Number of Events/Total in Intervention Group	Number of Events/Total in Control Group	Unadjusted OR (*p*-Value; 95%CI) Base on DL	Adjusted OR (*p*-Value; 95%CI) Base on GW and HK
(1) LPV/RTV vs. umifenovir	Li (2020, China) [19]; Wen (2020, China) [20]	54/86	39/58	0.87 (*p* = 0.70; 0.42, 1.78) (Figure 5 in [6])	0.43 (*p* = 0.14; 0.04, 4.92)
(2) LPV/RTV vs. no antiviral treatment (conventional)	Li (2020, China) [19]; Wen (2020, China) [20]	54/86	40/67	0.99 (*p* = 0.98; 0.49, 1.99) (Figure 6 in [6])	0.49 (*p* = 0.27; 0.01, 33.85)
(3) Rate of cough alleviation after 7 days of treatment (LPV/RTV vs. umifenovir)	Li (2020, China) [19]; Wen (2020, China) [20]	11/80	13/61	0.62 (*p* = 0.69; 0.66, 6.53) (Figure 7 in [6])	0.31 (*p* = 0.51; 0.0001, 1,082,807)
(4) Rate of cough alleviation after 7 days of treatment (LPV/RTV vs. no antiviral treatment)	Li (2020, China) [19]; Wen (2020, China) [20]	11/80	8/67	0.87 (*p* = 0.89; 0.10, 7.16) (Figure 8 in [6])	0.43 (*p* = 0.58; 0.0001, 417,569)
(5) Rate of improvement on chest CT after 7 days of treatment (LPV/RTV vs. umifenovir)	Li (2020, China) [19]; Wen (2020, China) [20]	32/87	29/69	0.80 (*p* = 0.5; 0.42, 1.54) (Figure 9 in [6])	0.40 (*p* = 0.13;0.04, 4.04)
(6) Rate of improvement on chest CT after 7 days of treatment (LPV/RTV vs. no antiviral treatment or conventional)	Li (2020, China) [19]; Wen (2020, China) [20]	32/87	34/74	0.69 (*p* = 0.26; 0.36, 1.31) (Figure 10 in [6])	0.34 (*p* = 0.14; 0.01, 8.11)
(7) Rate of adverse events of treatment (LPV/RTV vs. umifenovir)	Li (2020, China) [19]; Wen (2020, China) [20]; Jun (2020, China) [21]	45/145	15/105	2.66 (*p* = 0.004; 1.36, 5.19) (Figure 13 in [6])	0.83 (*p*= 0.60; 0.23, 3.03) (Refer to Figure 1 in this study)
(8) Rate of adverse events of treatment (LPV/RTV vs. no antiviral treatment or conventional)	Li (2020, China) [19]; Wen (2020, China) [20]; Jun (2020, China) [21]	45/145	10/123	4.6 (*p* = 0.0007; 1.91, 11.07) (Figure 14 in [6])	1.51 (*p* = 0.30; 0.42, 5.46) (Refer to Figure 2 in this study)

Abbreviations: DerSimonian-Laird, DL; Geographically weighted, GW; Hartung and Knapp, HK; Odds ratio, OR; Confidence interval, CI.

## Data Availability

The data that support the findings of this study are available in Table 1 in this article.

## Supplementary Materials

The following supporting information can be downloaded at: https://www.mdpi.com/article/10.3390/tropicalmed8020083/s1, Table S1: Relevant geographical characteristics of the studies.
